# Biochemical Characterization of the Split Class II Ribonucleotide Reductase from *Pseudomonas aeruginosa*

**DOI:** 10.1371/journal.pone.0134293

**Published:** 2015-07-30

**Authors:** Mikael Crona, Anders Hofer, Juan Astorga-Wells, Britt-Marie Sjöberg, Fredrik Tholander

**Affiliations:** 1 Department of Medical Biochemistry and Biophysics, Karolinska Institutet, Scheeles väg 2, SE-17177, Stockholm, Sweden; 2 Department of Medical Biochemistry and Biophysics, Umeå University, SE-90187, Umeå, Sweden; 3 Department of Biochemistry and Biophysics, Stockholm University, SE-10691, Stockholm, Sweden; University of Nottingham, UNITED KINGDOM

## Abstract

The opportunistic pathogen *Pseudomonas aeruginosa* can grow under both aerobic and anaerobic conditions. Its flexibility with respect to oxygen load is reflected by the fact that its genome encodes all three existing classes of ribonucleotides reductase (RNR): the oxygen-dependent class I RNR, the oxygen-indifferent class II RNR, and the oxygen-sensitive class III RNR. The *P*. *aeruginosa* class II RNR is expressed as two separate polypeptides (NrdJa and NrdJb), a unique example of a split RNR enzyme in a free-living organism. A split class II RNR is also found in a few closely related γ-Proteobacteria. We have characterized the *P*. *aeruginosa* class II RNR and show that both subunits are required for formation of a biologically functional enzyme that can sustain vitamin B12-dependent growth. Binding of the B12 coenzyme as well as substrate and allosteric effectors resides in the NrdJa subunit, whereas the NrdJb subunit mediates efficient reductive dithiol exchange during catalysis. A combination of activity assays and activity-independent methods like surface plasmon resonance and *gas* phase electrophoretic macromolecule analysis suggests that the enzymatically active form of the enzyme is a (NrdJa-NrdJb)_2_ homodimer of heterodimers, and a combination of hydrogen-deuterium exchange experiments and molecular modeling suggests a plausible region in NrdJa that interacts with NrdJb. Our detailed characterization of the split NrdJ from *P*. *aeruginosa* provides insight into the biochemical function of a unique enzyme known to have central roles in biofilm formation and anaerobic growth.

## Introduction

*P*. *aeruginosa* (PA) is an inherently drug resistant opportunistic pathogen and a major cause of severe nosocomial infections in immunocompromised patients, *e*.*g*. burn victims, cancer patients, and in cystic fibrosis patients [1, 2]. It is a facultative anaerobe with minimal nutritional requirements that can colonize and grow in a wide variety of environments. Its ability to proliferate regardless of the oxygen level is reflected by the fact that its genome encodes all three classes of ribonucleotide reductase (RNR), the sole enzyme capable of reduction of RNA building blocks (ribonucleotides) into DNA building blocks (deoxyribonucleotides).

The three classes of RNR are the oxygen dependent class I, the oxygen indifferent class II and the oxygen-sensitive class III; all sharing a common ancestry. The different classes use different cofactors for the generation of a catalytically essential radical. The canonical class I enzymes consist of a catalytic subunit and a radical harboring subunit possessing a dinuclear metal site [3]. These subunits assemble to form a functional class I enzyme, typically with an α_2_β_2_ quaternary structure. In contrast, class II consists of a single polypeptide, encoded by the *nrdJ* gene, and the functional enzyme is commonly monomeric or homodimeric [4, 5]. Class II RNRs utilize the B12 coenzyme adenosylcobalamin (AdoCbl) as a radical generator. Class III RNRs are homodimeric enzymes, which utilize a stable (but oxygen sensitive) glycyl radical in catalysis [6]. In the class III enzyme, radical formation requires a specific S-adenosylmethionine-dependent activase. Thus, only class II RNR contains the full machinery for radical generation and ribonucleotide reduction in a single polypeptide.

To maintain fidelity in DNA replication and repair, RNR enzymes have allosteric binding sites that assure a balanced production of dNTPs [7, 8]. All RNR classes possess a substrate specificity site that controls the selectivity for the four different substrates. In addition, almost all class I, and most class III RNRs, have an allosteric overall activity site acting as an on/off switch of the enzyme activity in response to ATP (on) and dATP (off). PA NrdJ, as well as more than 90% of all class II RNRs, lacks this latter allosteric site [9]. A common feature of class I and II RNR enzymes is that the flexible C-terminal tail of the catalytic subunit contains cysteine residues that mediate thiol-disulfide exchange at the active site after each catalytic turnover via interaction with the physiological reduction system.

Interestingly, almost all sequenced γ-Proteobacteria that encode class II RNR, including PA, harbor a unique subtype of the enzyme. The *nrdJ* gene in these species is split at a specific site into two genes, *nrdJa* and *nrdJb*, of a single operon that encodes the two polypeptides NrdJa (734 residues in PA) and NrdJb (229 residues in PA). A similar split in the *nrdJ* gene is also found in some β-Proteobacteria, and a few α- and ζ-Proteobacteria [4]. The NrdJ in these Proteobacteria is the only found instance of a split RNR in a non-viral organism.

A previous study showed that the *nrdJab* operon in PA is of functional relevance, *e*.*g*. its gene expression is increased in the stationary growth phase and when class I RNR is inactivated, and the operon is required to sustain vitamin B12-dependent growth [10, 11]. Moreover, deletion of the operon gives a phenotype with reduced virulence in a *Drosophila melanogaster* infection system, indicating that DNA precursor synthesis by PA NrdJ is important during infection [12]. Under anaerobic growth, NrdJ-coupled DNA precursor synthesis also appears to be crucial to achieve sufficient DNA replication for normal cell division of PA; without exogenous vitamin B12 cell elongation and biofilm formation occur [13]. NrdJ in PA thus has a key role for biofilm formation and growth.

Consequently, the unique split nature, involvement in virulence and central role in oxygen-independent DNA precursor synthesis, render NrdJa-NrdJb an interesting enzyme for detailed biochemical characterization. Our data show that the main function of NrdJb is to mediate disulfide exchange, while the catalytic activity and AdoCbl cofactor binding is confined to the NrdJa subunit. Allosteric effectors and substrate promote [NrdJa]_2_ formation and modulates the NrdJa-NrdJb interaction leading to a tight [NrdJa-NrdJb]_2_ complex. We have also mapped the binding location of NrdJb with respect to the NrdJa subunit. Finally, we show that the small NrdJb subunit is required for a functional enzyme *in vivo*.

## Results

### Expression and purification of NrdJa and NrdJb and initial screening of the reaction conditions

In a previous study the activity of the PA NrdJ enzyme was measured in crude extracts from PAO1 designed to overexpress NrdJa and NrdJb [10]. To allow a detailed biochemical characterization of the enzyme we now wanted to express and purify both subunits separately.

Expression and purification of NrdJa was readily performed as described in Materials and Methods. The final purity of NrdJa was >90%, as assessed by SDS-PAGE.

Initial attempts to express soluble NrdJb in *E*. *coli* BL21(DE3) failed due to formation of inclusion bodies. Therefore, we introduced codon optimization of the PA *nrdJb* gene for expression in *E*. *coli* and addition of a C-terminal 6xHis tag, which resulted in high yield expression (25 mg/l cell culture) and efficient purification of the protein to ~90% purity (as judged by SDS-PAGE) in a single chromatographic step. However, the protein had low solubility and precipitated at concentrations >3 mg/ml. Screening for better protein solubility [14] indicated that the protein kept soluble and was stable for long term storage in Tris buffer at pH>8. Thus, the whole purification protocol and final storage was performed at pH 8.3.

To identify initial reaction conditions for the activity assay, a two-level (pH was tested at three levels) fractional factorial screen was performed. The tested continuous factors were: pH (7/7.5/8), [dithiothreitol] (DTT) (30/100 mM), [tris(2-carboxyethyl)phosphine] (TCEP) (30/100 mM), [ATP] (0.75/1.5 mM), [AdoCbl] (25/100 μM), [MgCl_2_] (5/30 mM), and [NrdJb] (0/2 μM). [NrdJa] was always at 2 μM in all assays. In addition, substrate was tested as a categorical factor (*i*.*e*. a single concentration of CTP or CDP, 1 mM). The results revealed that CTP is the preferred substrate with a >60-fold higher activity compared to CDP, that the enzyme subunit ratio should be close to unity, and that DTT is preferred over TCEP as reducing agent. Interestingly, we observed that the NrdJa subunit alone had a small but measurable activity in presence of DTT (see further below). For ATP the effects could not be clearly resolved in these initial experiments, and for TCEP, MgCl_2_ and pH, the effects within the range tested were negligible. The results led to the following standard reaction mixture (as given in the methods section) that was used in subsequent experiments: 50 mM Tris-HCl buffer at pH 7.5, 30 mM DTT, 10 mM MgCl_2_, 100 μM AdoCbl, 1 mM ATP, 1 mM CTP, and 2 μM of NrdJa and NrdJb. Using these conditions the specific activity of the enzyme was determined to be 1.5 nmol/min/mg (with respect to the NrdJa subunit).

### Requirements for NrdJa-NrdJb enzyme activity

**Fig 1:**
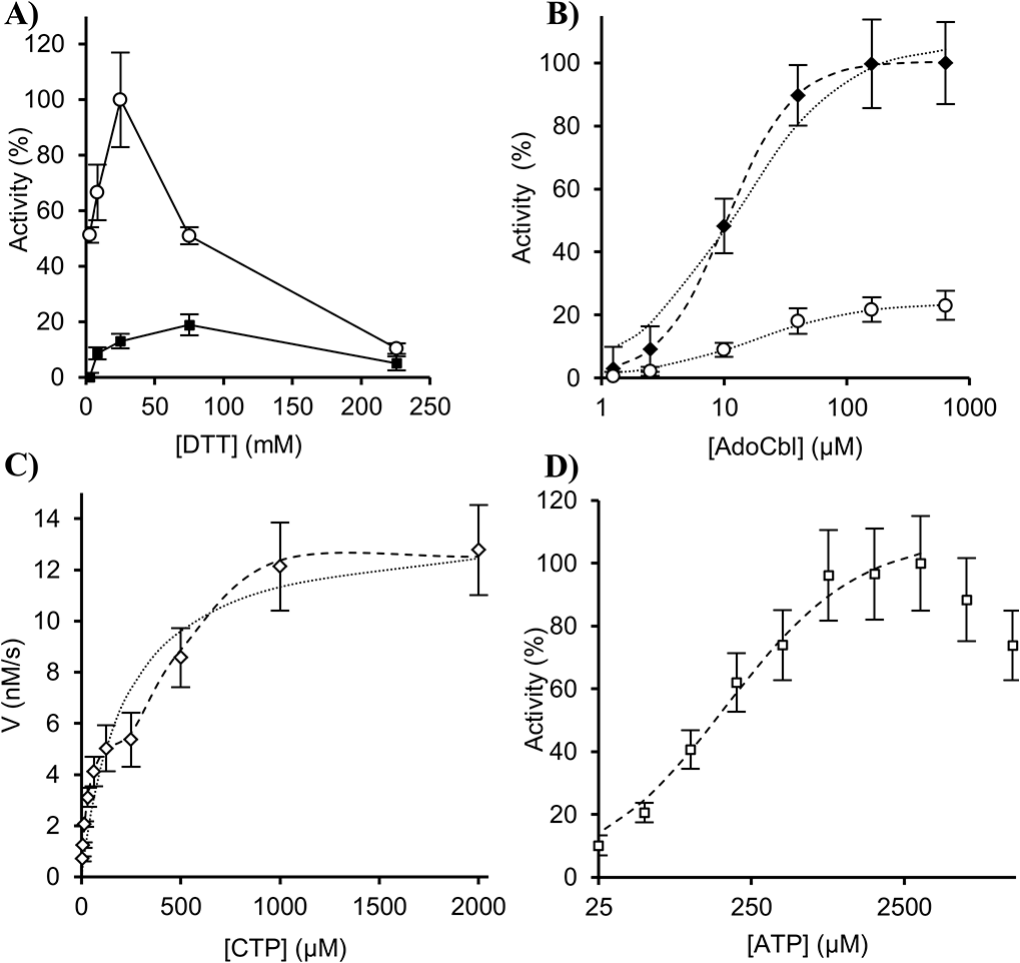
Enzyme activity experiments. Effects on enzyme activity of increasing concentrations of the reducing agent DTT (panel **A**), the cofactor AdoCbl (Panel **B**), the CTP substrate (panel **C**), and the allosteric effector ATP (panel **D**). Error bars indicate the standard deviation of three measurements. **A**) Enzyme activities of NrdJa-NrdJb (filled circles) and NrdJa (open circles) at saturating substrate concentration were measured with increasing concentration of DTT. **B**) Enzyme activities of NrdJa-NrdJb (black diamonds) and NrdJa (open circles) with increasing concentrations of AdoCbl and saturating substrate concentration were determined. Dotted lines correspond to a model for a single binding site fitted to the data and dashed line to a model for cooperative binding. **C**) The reaction velocity was determined with increasing concentration of CTP substrate. Models for classical Michaelis-Menten kinetics (dotted line), and a mixed model involving both Michaelis-Menten kinetics and Hill cooperativity (dashed line), were fitted to the data. **D**) Enzyme activity was determined at saturating substrate concentration with increasing concentrations of ATP. The fitted model curve used to derive the apparent *K*_d_ value is shown (dashed line).

Series of reaction mixtures, containing either NrdJa plus NrdJb (2 μM of each) or only NrdJa (2 μM), were titrated in the standard reaction with increasing amounts of DTT (2.8–256 mM) (**Fig 1A**). Whereas the activity of NrdJa-NrdJb peaked distinctly at a DTT concentration around 25 mM, NrdJa exhibited maximum activity at 75 mM DTT with a slow decline at higher concentrations.

The effect of increasing concentrations of AdoCbl on the enzyme activity was tested for both the NrdJa-NrdJb system and for NrdJa alone (**Fig 1B**) using 30 and 75 mM DTT in the standard reaction, respectively. The AdoCbl response saturated at concentrations above 50 μM and no significant difference in AdoCbl dependence was observed between NrdJa-NrdJb and NrdJa. A single-site binding model was fitted to the data (**Equation 1**) to derive apparent dissociation constants, *K*_0.5_ values. The determined *K*_0.5_ for AdoCbl was 13±4 μM for NrdJa-NrdJb and 17±4 μM for NrdJa. The small difference between these two values suggests that the NrdJb does not have a significant effect on the binding of AdoCbl. For the NrdJa-NrdJb-AdoCbl dataset, a model for cooperative binding (**Equation 2**) gave a better fit to the data, a weaker *K*_0.5_ (40±7 μM), and a Hill factor of 1.6±0.5.

To deduce kinetic parameters the reaction velocities were determined with increasing substrate concentrations, 3.9–2000 μM CTP, in the standard reaction (**Fig 1C**). Fitting the Michaelis-Menten model to the data gives a *K*_m_ of 220±60 μM and a *V*_max_ of 14±4 nM/s, but with a modest model-to-data fit (sum of squares 10, R^2^ 0.92). Since the reaction velocities suggested a biphasic substrate dependence with a second inflection point around 300 μM CTP, another kinetic model might be relevant. Such a kinetic behavior has been observed for enzyme mixtures acting on the same substrate, and for enzymes involving subunit interactions with strong cooperative effects [15]. As a comparison, a model that takes allosteric activation in response to increased substrate concentration was therefore also fitted to the data (**Equation 3)**. This model gives two *K*_m_ values, 18±2 μM and 460±20 μM, one *V*_max_ of 5.1±0.1 nM/s, a Hill factor of 6±2, and a better data fit (sum of squares 0.1, R^2^ 0.996). In this model, the total measured activity is the sum of two enzyme subsets, one that follows classical Michaelis-Menten kinetics, and one with Hill cooperativity. Since there is a risk of overfitting when using complex models, results based on this model should be interpreted cautiously. Nevertheless, it suggests that CTP has a positive cooperative effect.

The response in enzyme activity to increasing concentrations of ATP (0.025–12.8 mM) was analyzed at constant substrate concentration (1 mM CTP) (**Fig 1D**). The response was saturation-like up to 3–4 mM with a weak decline at concentrations above 5 mM. The latter effect is probably caused by competition between ATP and the assayed ^3^H-CTP substrate (note that ATP is both a substrate and an effector), thus causing an apparent inhibition. An apparent *K*_d_ value for ATP binding to the effector site was determined to 170±20 μM using a model for single site binding (**Equation 1**).

### NrdJa-NrdJb subunit interactions

**Fig 2:**
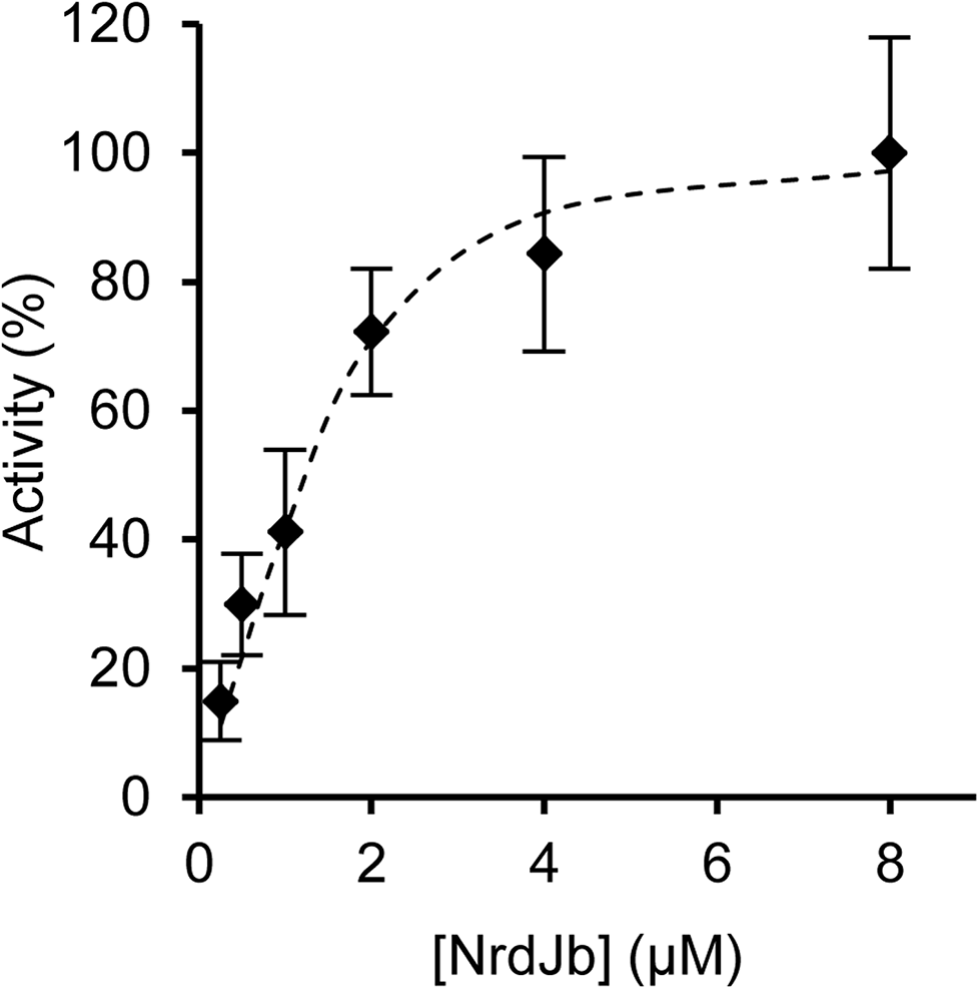
Enzyme activity with increasing [NrdJb]. Enzyme activity was determined at saturating concentration of substrate, constant concentration of NrdJa and increasing concentration of NrdJb. The apparent subunit dissociation constant was obtained by fitting a model with overall second order dependence on subunit concentrations to the data (dashed line).

To estimate the NrdJa-NrdJb subunit affinity, the effect of increasing concentrations of NrdJb (0.25–8 μM) was tested with constant concentration of NrdJa (2 μM) in the standard reaction (**Fig 2**). The linear increase in enzyme activity leveled off at approximately equimolar concentration of NrdJa and NrdJb. Fitting a model with overall second order dependence in subunit concentrations (**Equation 4**) gives an apparent *K*_d_ of 260±90 nM.

**Fig 3:**
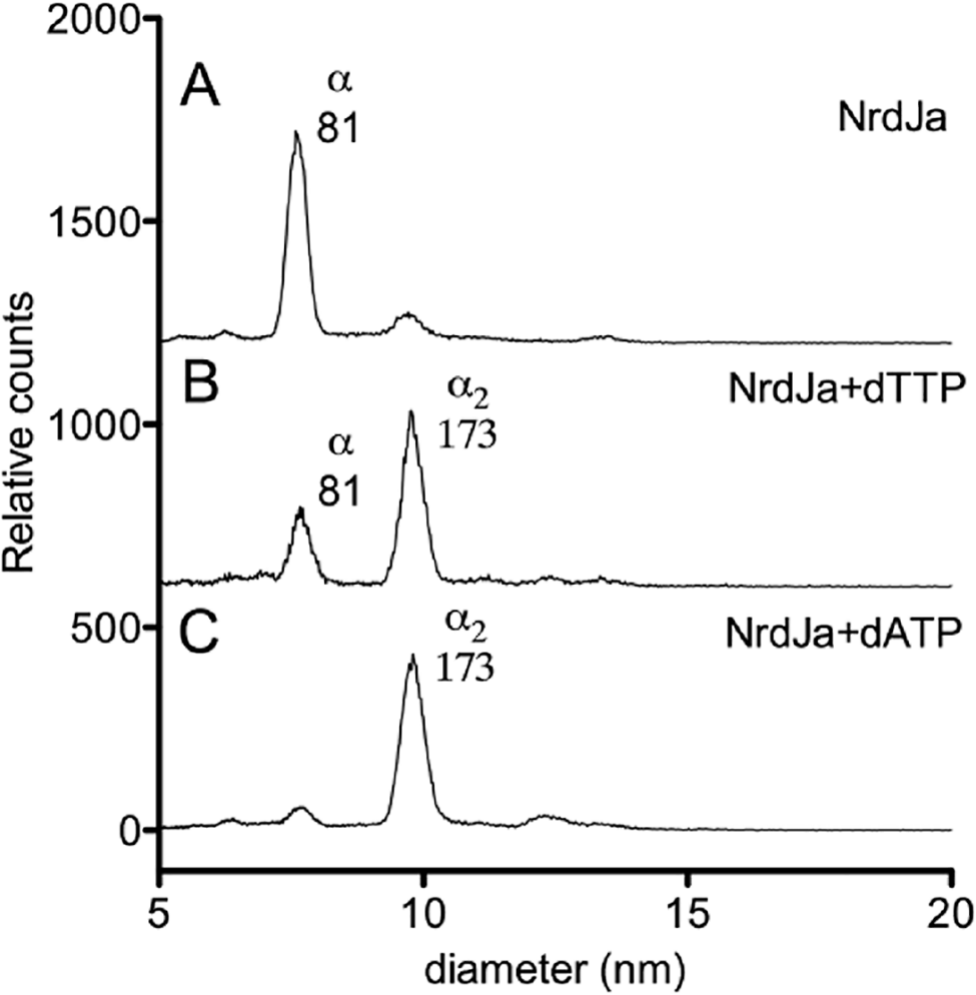
GEMMA analysis of protein oligomerization. Effectors induce protein dimerization of NrdJa. Without effector molecules, NrdJa is mostly in a monomeric state (81kDa, panel **A**). With dTTP (panel **B**) and dATP (panel **C**), the dimeric state dominates (173kDa).

To obtain a better understanding of factors that govern subunit interaction, a series of activity-independent experiments was performed. Gas phase electrophoretic macromolecule analysis (GEMMA) was used to determine the quaternary structure of NrdJa and NrdJb (**Fig 3**). In GEMMA, biomolecules are electrosprayed into gas phase, neutralized to singly charged particles, and the gas phase electrophoretic mobility is measured with a differential mobility analyzer. The mobility of an analyzed particle is proportional to its diameter, which therefore allows for quantitative analysis of the different particle sizes contained in a sample [16]. In the absence of allosteric effector, NrdJa was mainly present as monomer (81 kDa peak). Addition of the allosteric effector dTTP (50 μM), and more effectively dATP (50 μM), induced dimerization (173 kDa peak). NrdJb had low solubility in the GEMMA buffer and displayed a variety of presumed unspecific aggregation states. Surface Plasmon Resonance (SPR) was used to further explore the effect of CTP (0.5 mM), dATP (0.5 mM) and AdoCbl (40 μM) on the affinity between NrdJa and immobilized NrdJb (**Table 1 & S1 Fig**). The single component that had the most pronounced effect was dATP, which caused an 8-fold decrease of the apparent *K*_d_. While AdoCbl did not affect the subunit affinity, CTP caused a 50% reduction of the apparent *K*_d_ to a value comparable to that obtained in the activity assays. With all tested components present the apparent *K*_d_ was 54 nM, as compared to 690 nM for the subunits alone.

**Table 1:** *K*_d_ values from SPR analysis of the NrdJa-NrdJb interaction.

Additive	*K*_d_ (nM)
None	690±60
AdoCbl	680±100
CTP	360±90
dATP	91±17
CTP+AdoCbl	300±190
dATP+CTP+AdoCbl	54±12

Notably, the effect of CTP on the subunit interaction is in line with the cooperative effect of CTP on enzyme activity. Thus, at lower CTP concentrations the relative concentration of the NrdJa-NrdJb complex is lower, with the low intrinsic activity of NrdJa alone accounting for a larger proportion of the activity. At optimal effector and substrate concentrations, NrdJa-NrdJb complex formation probably facilitates access between the active site and the redox active cysteines at the C-terminal end of NrdJb, which in turn renders the enzyme more catalytically competent.

**Fig 4:**
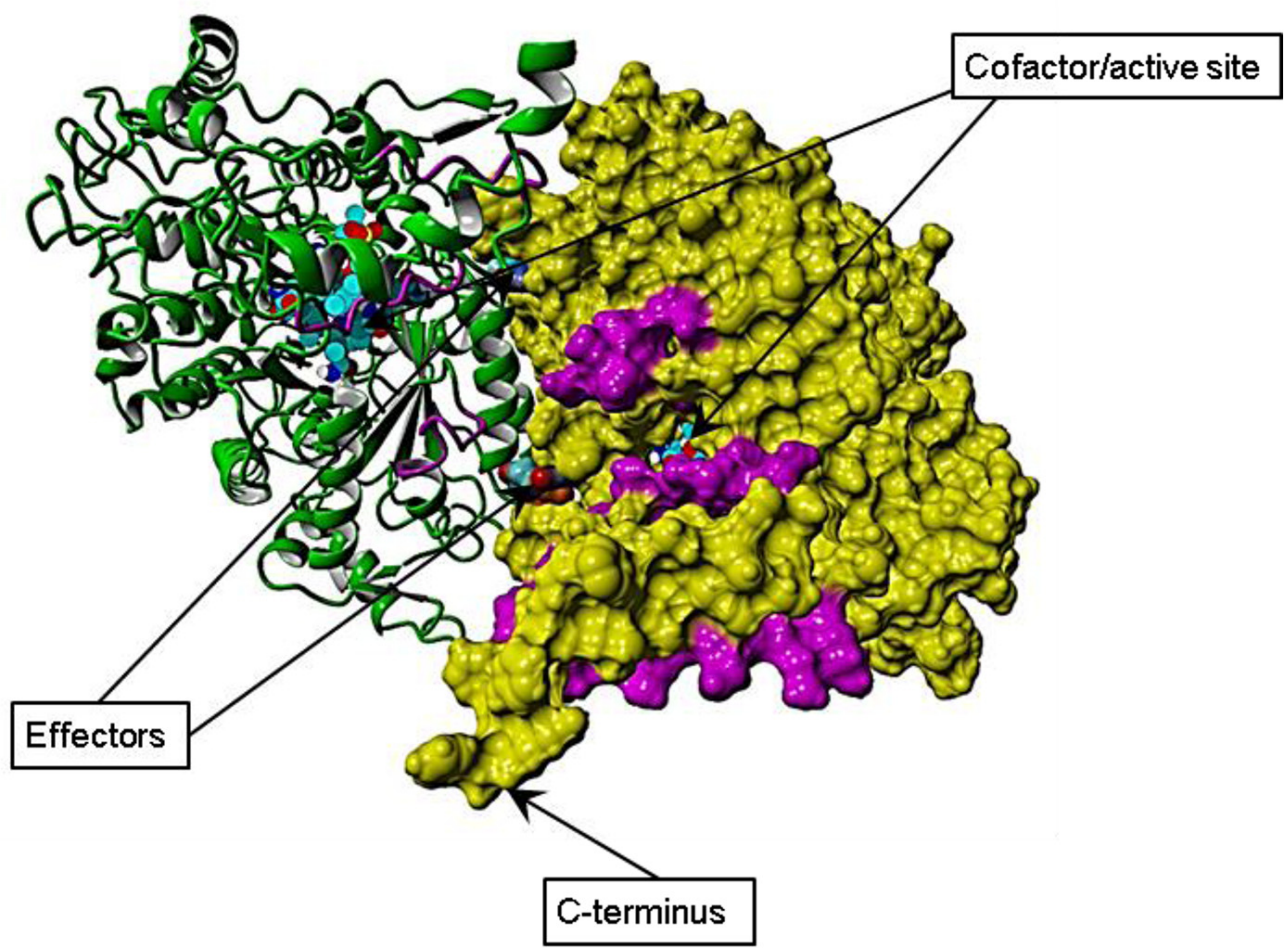
Model of NrdJa and HD exchange. The modeled NrdJa dimer is shown with one subunit in green ribbon representation, and the other subunit with the molecular surface in yellow. The C-terminus, the effector sites, and the cofactor and active sites are indicated. Areas exhibiting lowered deuterium exchange upon binding to NrdJb are indicated in magenta. From the model it is clear that AdoCbl and the active site clefts are confined to NrdJa. The NrdJb binding location is close to the C-terminal end of NrdJa and involves residues on the rim of the cofactor/active site cleft.

To obtain a three-dimensional model of the PA class II RNR, we used a combination of two different software solutions (YASARA and I-TASSER, as described in Materials and Methods). The NrdJb protein gave no hit to any template structure in the Brookhaven Protein Database. In contrast, the NrdJa protein sequence displayed reliable hits to several solved RNR structures. The derived model of NrdJa (**S1 File**) indicates that AdoCbl binding, effector sites, and substrate binding, are confined to NrdJa (**Fig 4 & S2 Fig**). The model shows a deep water-accessible cavity in which we can infer from the known RNR structures that the active site and the cofactor binding sites are closely located. In our model AdoCbl is surrounded by, and interacts with, structural elements of the NrdJa subunit. The last C-terminal residues in the model extend out from the protein suggesting that this is a flexible and possibly unordered part of the protein. This region is of importance because it pinpoints a likely binding position of the NrdJb protein. In an ancestral enzyme, NrdJa and NrdJb were presumably linked into a single NrdJ enzyme (as in other non-split NrdJ enzymes) and some features of the NrdJa-NrdJb interface are thus likely to be conserved from such an ancestral state.

The general fold of the NrdJa model was supported by the hydrogen-deuterium exchange (HDX) experiment with high exchange observed for peptides derived from loop regions and low exchange for central core regions (**S3 Fig**). Importantly, three peptides exhibited lower exchange upon addition of an excess of NrdJb (**Fig 4**), thus mapping its binding position.

### Essentiality of NrdJa and NrdJb

A previous study showed that AdoCbl can restore the growth of PA when class I RNR is inactivated by hydroxyurea (HU) [10]. In the same study, comparative enzyme assays with crude extracts from PA overexpressing either NrdJa, or NrdJa plus NrdJb, were interpreted to show that both subunits were required for class II RNR activity in the presence of HU, albeit NrdJa alone exhibited trace activity [10]. However, since the reductant DTT was used as an artificial electron donor for RNR, the roles of the separate NrdJa and NrdJb subunits were not assessed in a proper biological context.

**Fig 5:**
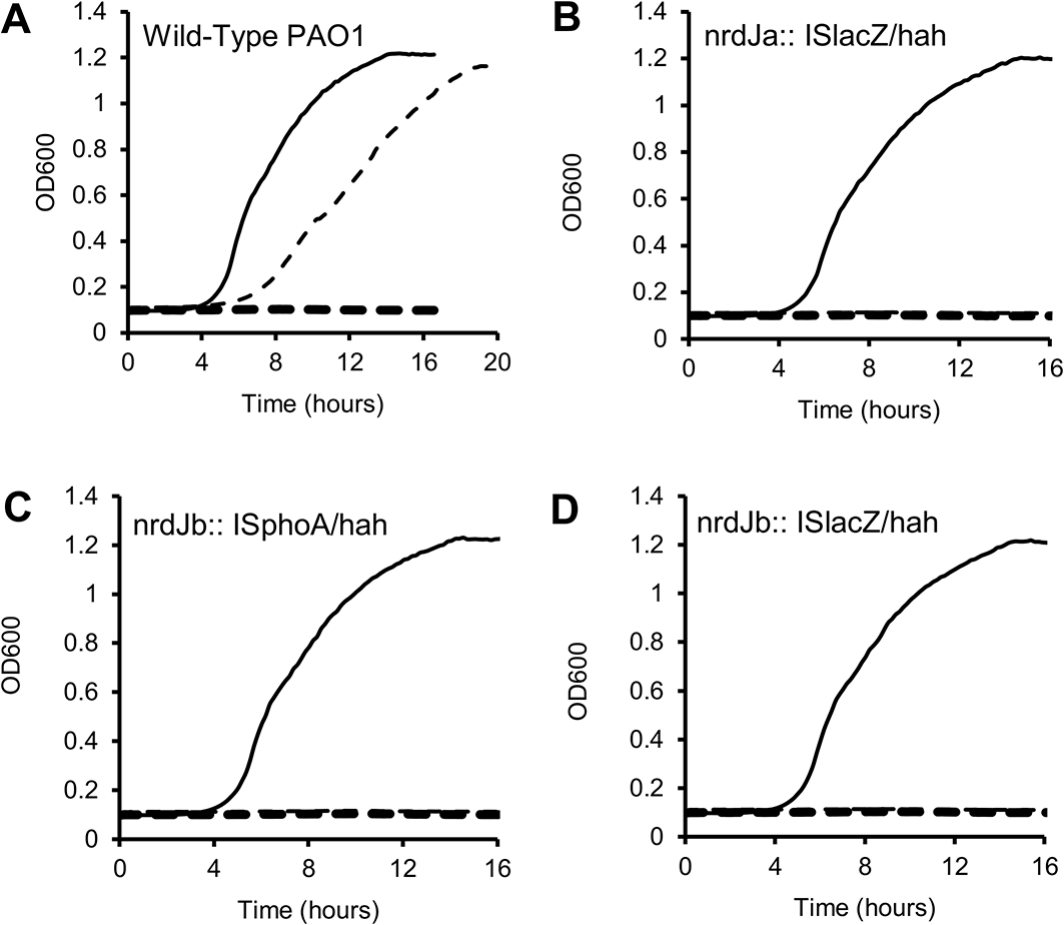
Bacterial growth curves. Wild-type (**A**), and three different *P*. *aeruginosa nrdJ* insertion mutants (**B**, nrdJa::ISlacZ/hah; **C**, nrdJb::ISphoA/hah; **D**, nrdJb::ISlacZ/hah) were cultivated in pure LB medium (black solid lines), in LB medium with HU (thick dashed lines), and in LB medium with HU and vitamin B12 (thin dashed lines). Mutant strains and wild-type exhibit the same growth pattern when grown in pure LB medium, but only wild-type could be rescued from the toxic effect of HU by addition of vitamin B12 (**A**, dashed line). Note that the dashed lines overlap in **B**-**D**.

To assess the specific roles of NrdJa and NrdJb separately, and in a biological context, we have therefore investigated if PA mutants with inserts in their *nrdJa* or *nrdJb* genes could support growth of PA under conditions with an impaired class I RNR. To this end, the growth of wild-type and PA mutants cultivated in parallel, and with different combinations of HU and AdoCbl in the growth medium, was monitored (**Fig 5**). HU (40 mM) served to inactivate class I RNR, and vitamin B12 (100 μM) to guarantee adequate cofactor supply for class II RNR.

Both the wild-type and mutant strains grew normally, with similar growth rates and reached similar OD_600_ values when grown in LB medium. When class I RNR was inhibited by addition of HU neither wild-type PA nor the mutants exhibited any signs of growth (OD_600_ values identical to sterile control medium) after 18 hours. On the other hand, when both HU and vitamin B12 were added to the medium the growth of wild-type PA was rescued and the culture reached saturation overnight (OD_600_ similar to controls with LB medium containing only vitamin B12). In contrast, none of the mutants could be rescued by addition of vitamin B12 and no bacterial growth (OD_600_ values identical to sterile control medium) was observed after cultivation overnight suggesting that both *nrdJa* and *nrdJb* are essential when class I RNR is impaired. The same results were observed with AdoCbl (data not shown) and cyanocobalamin (CNCbl), the common form of vitamin B12 in nutritional supplements), in line with the fact that CNCbl is converted to AdoCbl in the cell [17, 18].

## Discussion

### NrdJa and NrdJb have distinct mechanistic roles

Some class II RNRs are specific for ribonucleoside diphosphates as substrates, while other class II RNRs utilize only ribonucleoside triphosphates. For NrdJa-NrdJb from PA we found that NTP was the favored substrate. We also found that the separate NrdJa subunit had a small but measurable activity in a system using DTT as reducing agent (see below). The result shows that the full catalytic machinery for NTP reduction, including AdoCbl cofactor binding, resides within the NrdJa subunit, and that the main role of NrdJb is to provide thiol-disulfide exchange. The small DTT molecule can probably interact directly with the active site disulfide formed in NrdJa during catalysis to mediate its reduction, although less efficiently than the C-terminal cysteines in NrdJb. Similar results have been obtained for the C-terminal cysteine pair in *Escherichia coli* NrdA and *Lactobacillus leichmannii* NrdJ, demonstrating that non-physiological reducing agents indeed can reduce active site disulfides also in these RNRs [19–21]. The conclusion that NrdJb is responsible for thiol-disulfide exchange and that the active site and cofactor binding is confined to NrdJa, is supported by the similar AdoCbl-dependence of NrdJa-NrdJb and NrdJa. This is also in line with the SPR experiment where AdoCbl had marginal effects on the strength of the NrdJa-NrdJb complex, and the molecular modeling that suggests a binding site for AdoCbl in NrdJa.

The derived homology model indicates that the NrdJa subunit provides all structural elements to achieve cofactor, substrate and effector binding, thus supporting the results from the enzyme kinetic studies. The C-terminal part of the NrdJa model extends into the surrounding medium and pinpoints a plausible binding site for the NrdJb moiety. Interestingly, the observed shift in HDX upon NrdJb binding by NrdJa was localized to residues in the C-terminal region, and to spatially nearby residues around the rim of the cofactor-binding cavity. These residues are sufficiently close to the C-terminus to suggest that the relative binding position between NrdJa-NrdJb is partially conserved from an evolutionary single-chain ancestral protein. Together, this suggests that NrdJb binds close to the NrdJa C-terminal and close to the entrance of the cofactor-binding cleft. Such a binding mode is compatible with access to the active site by NrdJb and in line with its proposed role (this work) to mediate cysteine thiol-disulfide exchange.

### An ordered assembly of the active (NrdJa-NrdJb)_2_ complex

We have used a combination of experimental and theoretical methods to access the quaternary structure of the PA NrdJa-NrdJb complex. Enzyme activity assays demonstrated a 1:1 stoichiometry, GEMMA showed that NrdJa is a dimer in presence of allosteric effectors, and the SPR analyses demonstrated a more than 10-fold increase in the NrdJa-NrdJb affinity in the presence of allosteric effector and substrate. Notably, the SPR experiments were designed with NrdJa in solution and thus free to dimerize before binding to immobilized NrdJb, and the flexible nature of the SPR dextran-binding matrix generally permits gross complex formation and subunit interactions [22–24].Taken together, the GEMMA and SPR experiments therefore suggest that the ligand-induced increase of the interaction affinity is linked to dimerization.

The observed cooperative effects of substrate and AdoCbl in the activity assays are also in line with the GEMMA and SPR results, and further indicate that the active form of the enzyme is a dimer of dimers and that ligand binding facilitates oligomerization and catalysis. In addition, the HDX experiment combined with the GEMMA results and the stereochemical restrictions of the derived NrdJa model, strongly suggest that the PA class II RNR is a (NrdJa-NrdJb)_2_ homodimer of heterodimers.

We propose that formation of the PA class II RNR is initiated by dimerization of NrdJa in response to effector bound to the specificity site and substrate bound to the active site. As formation of the allosteric specificity sites at the subunit interface are directly linked to formation of the NrdJa dimer, ligand binding is a natural driving force to dimerization. The effector and substrate loaded NrdJa dimer is strongly primed to bind NrdJb, which enhances enzyme activity by efficient reduction of the active site cysteines in NrdJa. AdoCbl, with equal affinity for (NrdJa)_2_ and [NrdJa-NrdJb]_2_, probably binds after the substrate in order not to block access to the active site, as suggested by the structural architecture of the enzyme [25]. Along with the modest affinity for AdoCbl, this is in line with previous studies suggesting a catalytic mechanism involving cofactor release or relaxation in each catalytic cycle [25, 26]. This ordered arrangement of ligand and substrate loaded PA (NrdJa-NrdJb)_2_ assures optimal injection of the essential radical to the pre-assembled complex.

### Both NrdJa and NrdJb are essential *in vivo*

Previous findings indicate that the split PA NrdJ is biologically active; *e*.*g*. AdoCbl is required to rescue bacterial growth under conditions with an HU-inactivated class I RNR [10] and both genes of the *nrdJab* operon are expressed under certain growth conditions [13]. In addition, AdoCbl-dependent RNR activity was observed in DTT-supplemented crude extracts from PA overexpressing NrdJa and NrdJb, and it was concluded that PA class II RNR only shows activity when both NrdJa and NrdJb are present, even though trace enzyme activity was observed when only NrdJa was expressed [10].

Our current study of enzyme activity with purified enzyme confirmed that NrdJb is required for full enzymatic activity of NrdJa-NrdJb *in vitro*, but we could also demonstrate that the purified NrdJa protein possessed a low AdoCbl-dependent enzyme activity in presence of DTT.

To investigate if NrdJb is an essential component required for *in vivo* activity, we cultivated PA mutants with transposon inserts in *nrdJa* or *nrdJb* in medium supplemented with HU and vitamin B12. HU is bactericidal and acts by inactivation of the essential tyrosyl radical in class I RNR and by hydroxyl radical formation [27–29], and vitamin B12 is the required coenzyme of class II RNR (NrdJ). Notably, both HU-treatment and growth under anaerobic conditions give rise to an elongated cell morphology in PA that is attributable to hampered DNA synthesis [13, 30, 31], and PA does not have the capacity to circumvent hydroxyurea-mediated toxicity by endogenous AdoCbl production [10].

Our results clearly demonstrate that neither *nrdJa* nor *nrdJb* could be rescued from HU toxicity by addition of vitamin B12. Although this effect is in line with the phenotype observed when deleting the entire *nrdJab* operon [12], the HU- and AdoCbl-dependent effects of deleting the individual genes have not been assessed before. Thus, even though NrdJa alone has some trace activity *in vitro* with the artificial reducing agent DTT, its *in vivo* activity is dependent on a functional NrdJb protein that can mediate cysteine thiol-disulfide exchange via interaction with physiological redox systems.

### Concluding remarks

We have characterized the diverse functions of NrdJa and NrdJb components of the split PA class II RNR. The components bind tightly via interaction with residues around the rim of the AdoCbl-binding pocket, to form the active class II RNR *in vivo*. The catalytic machinery and cofactor binding is confined to the NrdJa subunit whereas the role of NrdJb is to mediate cysteine thiol-disulfide exchange. Allosteric effectors and substrate modulate the NrdJa-NrdJb interaction affinity mainly through dimerization of the NrdJa subunit and lead to formation of the (NrdJa-NrdJb)_2_ holoenzyme.

Species such as PA that encodes all three RNRs in their genome has an advantage as this provides flexibility with respect to the environmental oxygen level. This is particularly true for species encoding class II RNRs, which are indifferent to oxygen. Possible advantages associated with a split NrdJ enzyme are less obvious, but additional levels of regulation such as here described for the subunit interaction, or flexibility in the interaction with physiological reduction systems, are two possible examples.

The known RNR inhibitor HU is known to mediate its bactericidal effects by quenching the tyrosyl radical in class I RNR and by hydroxyl radical toxicity [29]. In class I RNRs, the radical is more exposed than in class II enzymes [25] and is also transported over a considerable distance. These observations, together with our finding that vitamin B12-mediated resistance to HU toxicity relies on an intact class II RNR, suggest that the class II RNR in PA might provide increased resistance to reactive oxygen species and fast adaptation between anaerobic and aerobic growth conditions. These are critical factors for the inherent flexibility and drug resistance of PA.

## Materials and Methods

### Materials

All standard chemicals were from Sigma Aldrich, Sweden. PA O1 strains with mutations in NrdJa (nrdJa-G07:: ISlacZ/hah) or NrdJb (nrdJb:: ISlacZ/hah and nrdJb:: ISphoA/hah) were from The *Pseudomonas aeruginosa* PAO1 transposon mutant library, the University of Washington, USA. These mutants correspond to strain PW10295 (genotype nrdJb-A12:: ISlacZ/hah), PW10296 (genotype nrdJb-F12:: ISphoA/hah), and PW10298 (genotype nrdJa-G07:: ISlacZ/hah), respectively, in the mutant collection library and has transposon inserts in the indicated genes [32]. The gene encoding NrdJb with an N-terminal 6xHis-tag was custom synthesized with codon optimization for expression in *E*. *coli* and cloned into pET21 by Epoch Life Science, Inc.

### Expression and purification

The *nrdJa* open reading frame cloned into the pET22 vector was expressed in *E*. *coli* BL21(DE3) and purified by ammonium sulfate precipitation, hydrophobic interaction chromatography, and anion exchange chromatography, basically following the procedures described previously [33]. The following modifications to the previously described methods were done: ampicillin (50 μg/ml) was used in the growth medium, 250 μM isopropyl-thio-D-galactopyranoside was used for induction, and cells were lysed by sonication.

For NrdJb, *E*. *coli* BL21(DE3) was transformed with the NrdJb construct (see materials) and grown in LB medium (30ml) containing 100 μg/ml carbenicillin at 37°C overnight. The overnight culture was inoculated into 1L LB medium containing 100 μg/ml carbenicillin and incubated under shaking at 37°C until an OD_600_ of 0.4 was reached. Expression of recombinant protein was induced with addition of 100 μM isopropyl-thio-D-galactopyranoside and incubation for 4 hours at 37°C. Cells were harvested by centrifugation for 10 minutes at 5000 g, resuspended in ice-cold lysis buffer (50 mM Tris-HCl pH 8.3, 10 mM imidazole, 300 mM NaCl, and 1 mM phenylmethylsulfonyl fluoride), and lysed by sonication. The lysate was centrifuged at 10.000 g at 4°C for 20 min to remove insoluble material. The resulting supernatant was mixed with ~1.5 ml Ni-loaded Chelating Sepharose (GE Healthcare), and then incubated under stirring for 10 min at 4°C. Subsequently, the lysate-resin mixture was poured into a column and washed with 10 ml of 20 mM Tris-HCl pH 8.3, containing 50 mM imidazole and 500 mM NaCl. His-tagged NrdJb was eluted with 10 ml 20 mM Tris-HCl pH 8.3, containing 250 mM imidazole and 500 mM NaCl. Eluted protein was immediately desalted on a gel filtration column (PD-10, GE Healthcare), concentrated and stored at -70°C in 20 mM Tris-HCl pH 8.3, supplemented with 10 mM TCEP.

### Enzyme activity assays

Enzyme assays were performed in 50 mM Tris-HCl at pH 7.5 with ^3^H-labelled CTP as substrate in volumes of 50 μl. In a standard reaction the constituents were; 30 mM DTT, 10 mM MgCl_2_, 100 μM AdoCbl, 1 mM ATP, 1 mM CTP (or when indicated CDP), and 2 μM of NrdJa and NrdJb. Some components were explicitly varied in specific experiments, see results section for details. Substrate was added last to start the reactions. Enzyme reactions were incubated for 45 minutes and then stopped by boiling. The chosen incubation time gave a maximum substrate turnover of <20%. The formed deoxyribonucleotide product was separated from ribonucleotides using boronate affinity chromatography using Affi-Gel Boronate Gel, or Dowex-50 ion exchange chromatography resin, and quantified with liquid scintillation counting, as described [34–36].

### GEMMA analysis

The GEMMA instrumental setup and general procedures were as described previously [24]. For NrdJa experiments, 40 mM ammonium acetate pH 7.8, 0.005% (v/v) Tween-20, 2 mM DTT and 0.02 mg/ml NrdJa was used. Effector nucleotides 50 μM dTTP or dATP with 1:1 Mg^2+^ were added as specified. For NrdJb experiments, 100 mM ammonium acetate pH 7.8, 0.005% (v/v) Tween-20, with or without 10 mM 2-mercaptoethanol, and variable amounts of NrdJb (0.027–0.068 mg/ml) was used.

### Surface plasmon resonance

PA NrdJa-NrdJb protein interaction analysis was in general performed as described previously [23]. Biotinylation with two molar excess biotin linker resulted in 0.15 biotin linker/NrdJa monomer, and in 0.05 biotin linker/NrdJb monomer. Both NrdJa and NrdJb proteins could be immobilized to a high degree onto streptavidin sensor chips (3665 and 2400 Response Units, respectively). Injected NrdJa could interact with immobilized NrdJb and vice versa, but due to limited stability of immobilized NrdJa during washing conditions and high unspecific binding of injected NrdJb we preferred to use immobilized NrdJb onto the sensor chip and injected NrdJa in the quantitative studies. After screening of binding conditions we decided to use the following injection conditions: 30 mM Tris-HCl pH 7.0, 10 mM MgCl_2_, 0.025% P20, 2 mM DTT and 0–2 μM NrdJa protein. Additional substrate, effector and cofactor were as described in specific experiments. Regeneration conditions of the binding surface was tested, and a combination of glycine pH 1.5–2.0 followed by injection of 8 M urea removed the majority of the bound NrdJa protein without destroying the immobilized NrdJb protein. Injection at 30 μl/min of NrdJa at 6 different concentrations was performed for all conditions, and all binding experiments were performed at least in duplicates. NrdJa-NrdJb interaction affinity was calculated from steady state data using the Michaelis-Menten equation.

### Molecular modeling

To obtain a 3D model of the NrdJa protein a combination of two different software solutions were used, YASARA and the I-TASSER server. YASARA [37] was used for homology modeling of the part of the NrdJa sequence for which reliable protein structure templates (3O0Q, 2WGH, 3TB9, 3K8T, 3HNC and 2XAP) of high quality and sequence similarity could be identified (software parameters set at default values). I-TASSER [38, 39], which uses a combination of threading and *ab initio* modeling, was used to predict the structure of the whole sequence of NrdJa (structural threading templates used: 2WGH, 1ZYZ, 3O0Q, 1XJE). The finally obtained top ranked model of each of the two approaches was very similar in the parts that could be modeled by both methods and could thus be combined into a single full length model. The combined model was subsequently refined in explicit solvent with YASARA by steepest descent minimization and simulated annealing, using the YASARA2 force field with periodic boundary conditions and a simulation cell extending 8 Å from the protein, until convergence (ΔE< 0.05 kJ/mol per atom per 200 steps) was reached. Prior to minimization, the hydrogen bonding network was optimized [40], pKa values assigned, and the simulation cell neutralized with salt ions (NaCl 0.9%) [41]. Ligand binding was also taken into account and deduced by superpositioning with structural templates with bound ligands.

### Hydrogen-deuterium exchange mass spectrometry analysis to map the NrdJb binding by NrdJa

Amide HDX analysis of the NrdJa and NrdJa-NrdJb complex was carried by mixing either the NrdJa alone or NrdJa-NrdJb complex with a deuterated buffer having the same ionic composition as the protein sample. To prepare a stock solution for studying NrdJa alone, 4 μl of 54.5 mg/ml NrdJa in 25 mM Tris-HCl pH 7.5 was mixed with 47 μl of a solution containing 1 mM ATP, 1 mM CTP and 10 mM magnesium acetate in 50 mM Tris-HCl pH 8.0 and 4 μl of 50 mM Tris-HCl pH 8.0. To prepare a stock solution for studying the NrdJa-NrdJb complex, 4 μl of 54.5 mg/ml NrdJa in 25 mM Tris-HCl pH 7.5 was mixed with 47 μl of a solution containing 1 mM ATP, 1 mM CTP and 10 mM magnesium acetate in 50 mM Tris-HCl pH 8.0, followed by the addition of 4 μl of 40 mg/ml NrdJb in 50 mM Tris-HCl pH 8.0. The deuteration reaction was started by mixing 3.3 μl of the NrdJa stock solution (or NrdJa-NrdJb stock solution) with 17 μl of deuterated buffer having the same ionic composition and pH as the sample. After 100 sec of incubation in deuterated buffer, each reaction was stopped by adding 35 μl of ice-cold quenching solution (2 M urea, 125 mM TCEP and 100 mM phosphate buffer pH 2.3) and immediately frozen into liquid N_2_. Samples were analyzed in a semi-automated HDX-MS system (Biomotif AB, Sweden) in which manually injected samples were automatically digested, cleaned and separated at 1.0°C. Briefly, samples were digested for 1 min using an in-house packed immobilized pepsin column with a flow rate of 50 μL/min, followed by an online desalting step with a 2.0 x 10 mm C-18 precolumn (ACE HPLC Columns, UK) using 0.05% TFA at 400μl/min for 2.5 min, and further separated by a C-18 Halo 2.1 x 100 mm column (Advance Materials Technology, USA) using a linear gradient of acetonitrile (5–40% in 8.5 min) in 0.3% formic acid using a flow rate of 75 μL/min. An Orbitrap XL mass spectrometer (ThermoScientific, USA) operated at 60k resolution was utilized for the analysis of deuterated samples. Mascot Search Engine (Matrix Science) was used to identify the peptic peptides derived from the digestion of NrdJa. HDExaminer Software (Sierra Analytics, USA) was used to process all HDX MS data.

### Bacterial cultivation

Wild-type and different *nrdJ* mutants (see materials) of *P*. *aeruginosa* PAO1 were used to study the effect of 40 mM HU on bacterial growth in LB medium (10 g tryptone, 5 g yeast extract, 5 g NaCl, and 1 mmol NaOH per liter) and in LB medium supplemented with 100 μM AdoCbl or CNCbl. Overnight cultures grown in LB medium were diluted 25-fold in fresh medium and grown until the OD_600_ reached ~0.5 and then diluted 500-fold and placed on ice. For each bacterial strain, 500 μl (corresponding to a ~0.25 cm path length in OD_600_ measurements with the used microwell plate) cultures supplemented with HU, HU + vitamin B12, or cultures with only LB medium were then immediately prepared and grown in the wells of a 24-well cell culture plate. Cell growth was monitored by measuring the OD_600_ with a microplate reader. All cultivation was performed at 37°C with gentle shaking in the dark.

### Model fitting and data analysis

Model-to-data fitting was performed with non-linear regression using the Solver add-in bundled with excel [42]. Solverstat was used to analyze model-to-data fit and to derive the reported standard errors of deduced model parameters [43]. The following equations were used in the model fitting:

**Equation 1**. *Activity* = ([*C*]·*A*_max_) ∕ (*K*_0.5_+[*C*]); were [*C*] denotes the analyte concentration, *A*_max_ the maximum activity, and *K*_0.5_ the apparent concentration giving half-maximum response

**Equation 2.** *Activity* = ([*C*]^h^·*A*_max_) ∕ (*K*_0.5_+[*C*]^h^); were *h* denotes the Hill factor, other notations as in equation 1.

**Equation 3.** *v*_o_ = ([*S*]^h^·*V*_max_) ∕ (*K*_m1_+[*S*])+([*S*]^h^·*V*_max_) ∕ (*K*_m2_+[*S*]^h^); were *v*_o_ denotes initial velocity, *V*_max_ maximum reaction velocity, [*S*] substrate concentration, *K*_m1_ the classical Michaelis constant, and *K*_m2_ the Michaelis constant for the enzyme subset with Hill cooperativity, and *h* the Hill factor.

**Equation 4.** *v* = *k* (0.5 [*NrdJa*]+[*NrdJb*]+*K*_d_)−0.5 √ (([*NrdJa*]+[*NrdJb*]+*K*_d_)^2^−4 [*NrdJa*] [*NrdJb*]))

Since the reactions were performed at a saturating concentration of substrate, the reaction velocity is independent of [*S*] and thus governed by the concentration of [*NrdJa-NrdJb*]. Hence, *v* = *k* [NrdJa-NrdJb], were [NrdJa-NrdJb] depends on [NrdJa] and [NrdJb] as shown in the equation.

## Supporting Information

S1 Fig SPR analysis of the NrdJa-NrdJb interaction. Surface plasmon resonance data of 0–2 μM NrdJa injected over immobilized NrdJb proteins in the absence (**A**) or presence of 40 μM AdoCbl (**B**), 0.5 mM CTP (**C**), 0.5 mM CTP + 40 μM AdoCbl (**D**), 0.5 mM dATP (**E**) and 40 μM AdoCbl + 0.5 mM CTP + 0.5 mM dATP (**F**). Sensorgrams showing binding in response units (RU) on the Y-axis with time after injection on the X-axis are displayed on the left side and corresponding steady state affinity plots fitted with the Michaelis-Menten equation on the right side.
(PDF)

S2 Fig Primary structure of NrdJa. The primary structure of NrdJa with secondary structure elements and residues in contact with ligands indicated (*G*, *T* and *B* denote residues in contact with the GTP substrate, the TTP effector and the AdoCbl co-factor, respectively). β-turns (β), γ-turns (γ), and β-hairpins (red horizontal ∩-lines) are denoted. Helices are numbered in the order of appearance (H1-H37) and strands by their designated sheets (A-G) The figure was generated with PDBsum (http://www.ebi.ac.uk/pdbsum/) with the derived model of NrdJa as input.
(PDF)

S3 Fig Single time-point deuteration profile of NrdJa by HDX MS analysis. A) Identified peptides derived from pepsin digestion of NrdJa are depicted with horizontal green lines above the NrdJa protein sequence, and the corresponding color-coded deuteration level (see key inset) is given below the sequence. Peptides exhibiting decreased deuteration upon NrdJb binding are boxed (215–225, 682–694, 702–708 and 702–709). **B**) The deuteration level mapped onto a ribbon representation of the model of the NrdJa dimer. The HDX MS data was in general agreement with the predicted structure of the NrdJa dimer.
(PDF)

S1 File NrdJa model. ZIP archive of PDB file with coordinates for the NrdJa model. The file contains the structure of the modelled NrdJa dimer with bound ligands (GTP substrate, TTP effector in complex with Mg^2+^, and AdoCbl). Solvent molecules used during modelling have been removed for clarity.
(ZIP)
