# Supplementary material for: Biochemical Characterization of the Split Class II Ribonucleotide Reductase from Pseudomonas aeruginosa
Source: PLoS One. 2015 Jul 30;10(7):e0134293. doi: 10.1371/journal.pone.0134293 (PMC4520616; doi:10.1371/journal.pone.0134293)
Supplement: S1 Fig — Surface plasmon resonance data of 0–2 μM NrdJa injected over immobilized NrdJb proteins in the absence (A) or presence of 40 μM AdoCbl (B), 0.5 mM CTP (C), 0.5 mM CTP + 40 μM AdoCbl (D), 0.5 mM dATP (E) and 40 μM AdoCbl + 0.5 mM CTP + 0.5 mM dATP (F). Sensorgrams showing binding in response units (RU) on the Y-axis with time after injection on the X-axis are displayed on the left side and corresponding steady state affinity plots fitted with the Michaelis-Menten equation on the right side. (PDF) [file pone.0134293.s001.pdf]

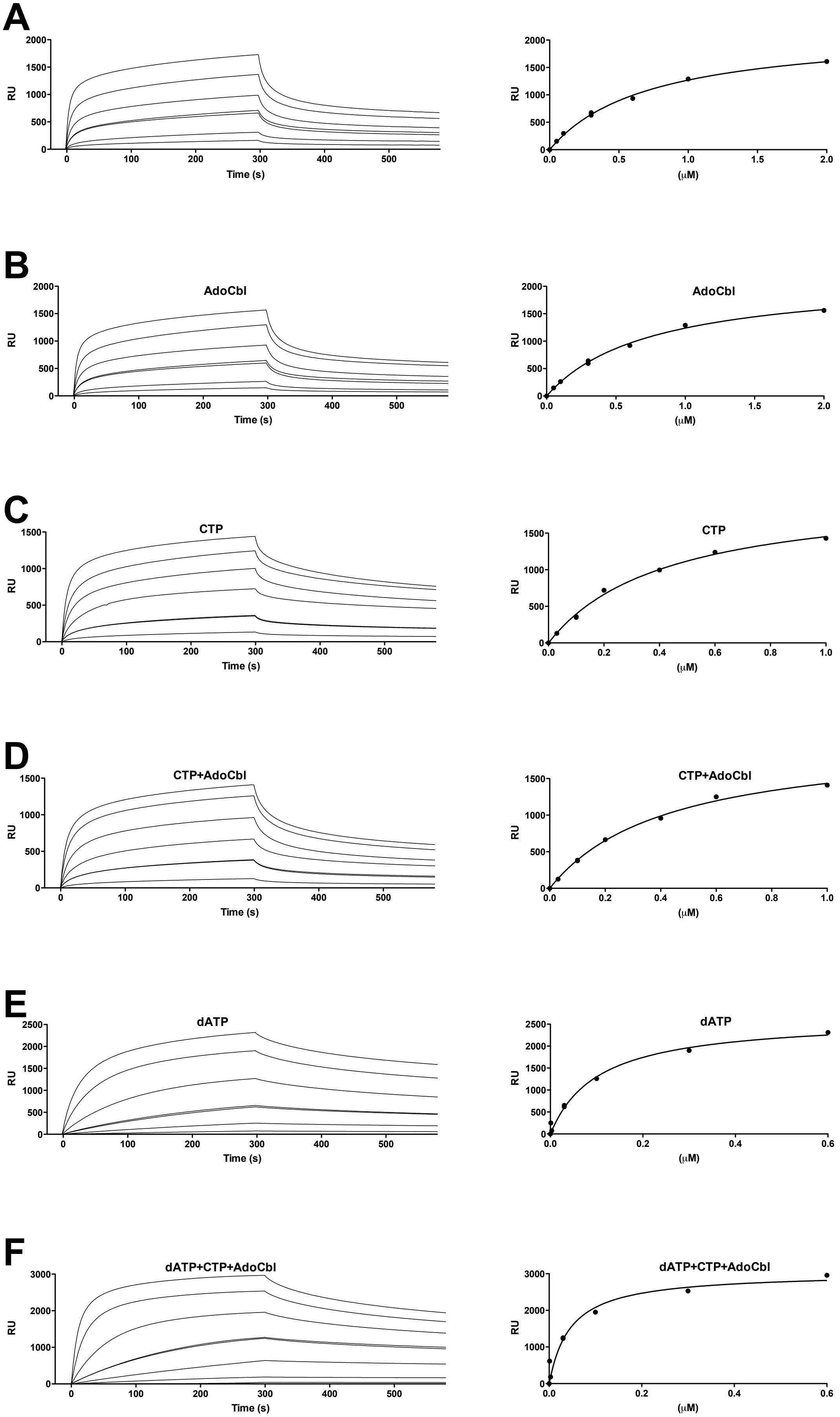
A
RU
Time (s)
RU
(μM)
B
AdoCbl
RU
Time (s)
AdoCbl
RU
(μM)
C
CTP
RU
Time (s)
CTP
RU
(μM)
D
CTP+AdoCbl
RU
Time (s)
CTP+AdoCbl
RU
(μM)
E
dATP
RU
Time (s)
dATP
RU
(μM)
F
dATP+CTP+AdoCbl
RU
Time (s)
dATP+CTP+AdoCbl
RU
(μM)

S1 Figure. SPR analysis of the NrdJa-NrdJb interaction.

Surface plasmon resonance data of 0-2 µM NrdJa injected over immobilized NrdJb proteins in the absence (A) or presence of 40 µM AdoCbl (B), 0.5 mM CTP (C), 0.5 mM CTP + 40 µM AdoCbl (D), 0.5 mM dATP (E) and 40 µM AdoCbl + 0.5 mM CTP + 0.5 mM dATP (F). Sensorgrams showing binding in response units (RU) on the Y-axis with time after injection on the X-axis are displayed on the left side and corresponding steady state affinity plots fitted with the Michaelis-Menten equation on the right side.
