# Supplementary material for: Biochemical Characterization of the Split Class II Ribonucleotide Reductase from Pseudomonas aeruginosa
Source: PLoS One. 2015 Jul 30;10(7):e0134293. doi: 10.1371/journal.pone.0134293 (PMC4520616; doi:10.1371/journal.pone.0134293)
Supplement: S2 Fig — The primary structure of NrdJa with secondary structure elements and residues in contact with ligands indicated (G, T and B denote residues in contact with the GTP substrate, the TTP effector and the AdoCbl co-factor, respectively). β-turns (β), γ-turns (γ), and β-hairpins (red horizontal ∩-lines) are denoted. Helices are numbered in the order of appearance (H1-H37) and strands by their designated sheets (A-G) The figure was generated with PDBsum (http://www.ebi.ac.uk/pdbsum/) with the derived model of NrdJa as input. (PDF) [file pone.0134293.s002.pdf]

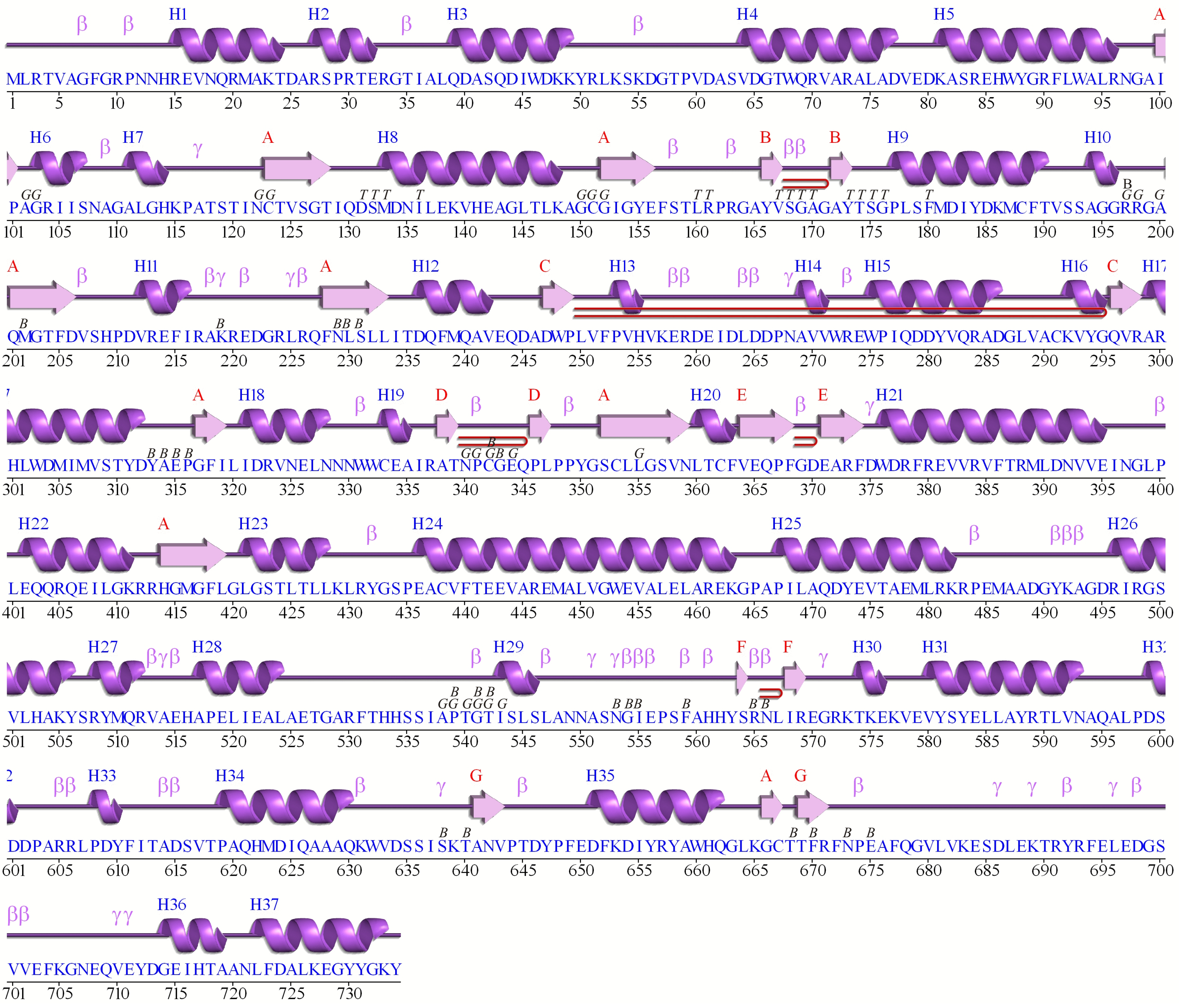

S2 Figure. Primary structure of NrdJa. The primary structure of NrdJa with secondary structure elements and residues in contact with ligands indicated (G, T and B denote residues in contact with the GTP substrate, the TTP effector and the AdoCbl co-factor, respectively). β-turns (β), γ-turns (γ), and β-hairpins (red horizontal ∩-lines) are denoted. Helices are numbered in the order of appearance (H1-H37)and strands by their designated sheets (A-G) The figure was generated with PDBsum (http://www.ebi.ac.uk/pdbsum/) with the derived model of NrdJa as input.
