# Supplementary material for: Biochemical Characterization of the Split Class II Ribonucleotide Reductase from Pseudomonas aeruginosa
Source: PLoS One. 2015 Jul 30;10(7):e0134293. doi: 10.1371/journal.pone.0134293 (PMC4520616; doi:10.1371/journal.pone.0134293)
Supplement: S3 Fig — A) Identified peptides derived from pepsin digestion of NrdJa are depicted with horizontal green lines above the NrdJa protein sequence, and the corresponding color-coded deuteration level (see key inset) is given below the sequence. Peptides exhibiting decreased deuteration upon NrdJb binding are boxed (215–225, 682–694, 702–708 and 702–709). B) The deuteration level mapped onto a ribbon representation of the model of the NrdJa dimer. The HDX MS data was in general agreement with the predicted structure of the NrdJa dimer. (PDF) [file pone.0134293.s003.pdf]

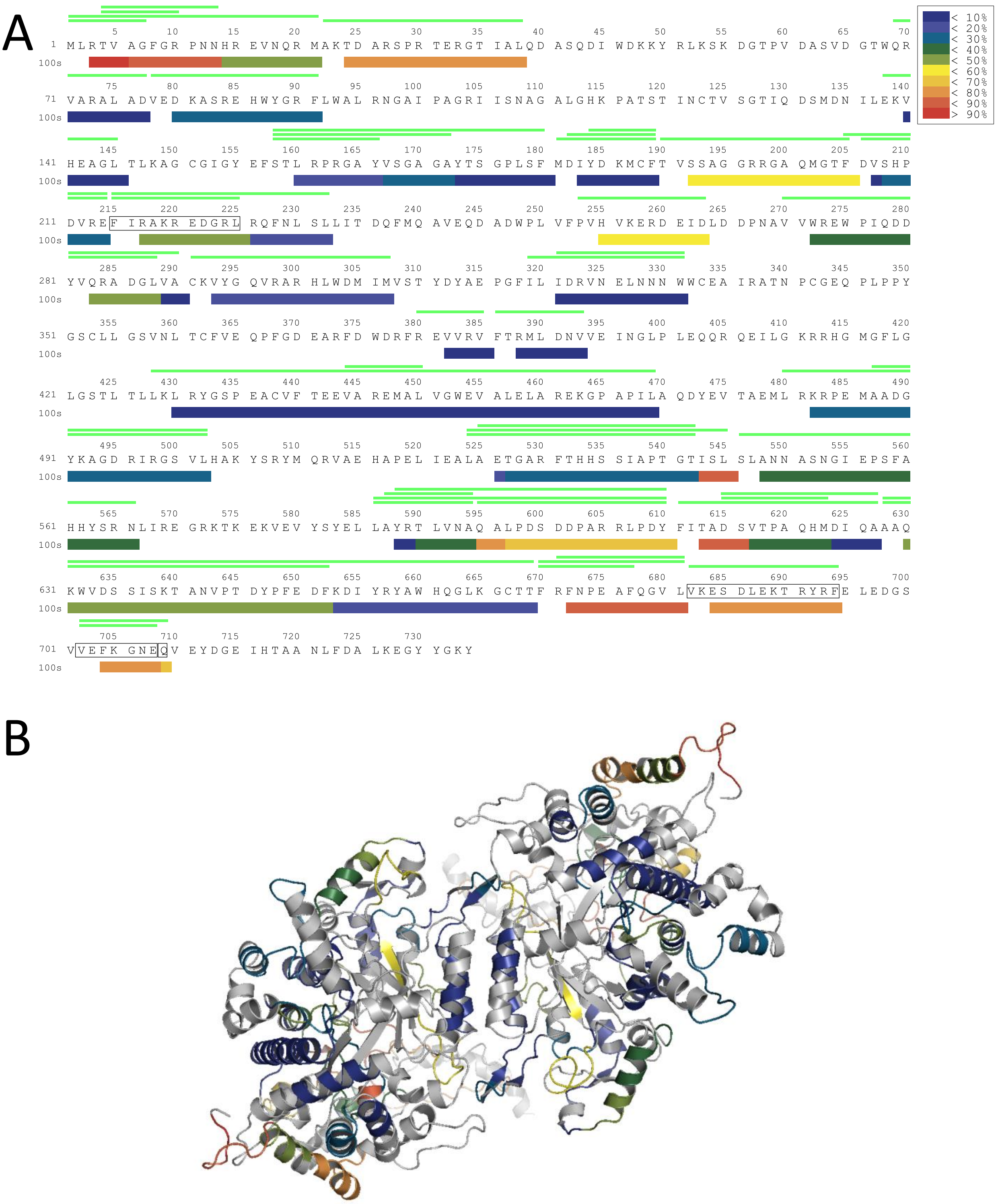

S3 Fig. Single time-point deuteration profile of NrdJa by HDX MS analysis. A)Identified peptides derived from pepsin digestion of NrdJa are depicted with horizontal green lines above the NrdJa protein sequence, and the corresponding color-coded deuteration level (see key inset) is given below the sequence. Peptides exhibiting decreased deuteration upon NrdJb binding are boxed (215-225, 682-694, 702-708 and 702-709). B) The deuteration level mapped onto a ribbon representation of the model of the NrdJa dimer. The HDX MS data was in general agreement with the predicted structure of the NrdJa dimer.
